# The Study of Mechanisms of Protective Effect of Rg1 against Arthritis by Inhibiting Osteoclast Differentiation and Maturation in CIA Mice

**DOI:** 10.1155/2014/305071

**Published:** 2014-08-21

**Authors:** Yanqing Gu, Weimin Fan, Guoyong Yin

**Affiliations:** Department of Orthopedics, The First Affiliated Hospital of Nanjing Medical University, 300 Guang Zhou Road, Nanjing 210000, China

## Abstract

Ginsenoside Rg1 is a natural product extracted from* Panax ginseng* C.A. Although Rg1 protects tissue structure and functions by inhibiting local inflammatory reaction, the mechanism remains poorly understood. *In vitro*, Rg1 dose-dependently inhibited TRAP activity in receptor activator of nuclear factor-*κ*B ligand- (RANKL-) induced osteoclasts and decreased the number of osteoclasts and osteoclast resorption area. Rg1 also significantly inhibited the RANK signaling pathway, including suppressing the expression of Trap, cathepsin K, matrix metalloproteinase 9 (MMP9), and calcitonin receptor (CTR). *In vivo*, Rg1 dramatically decreased arthritis scores in CIA mice and effectively controlled symptoms of inflammatory arthritis. Pathologic analysis demonstrated that Rg1 significantly attenuated pathological changes in CIA mice. Pronounced reduction in synovial hyperplasia and inflammatory cell invasion were observed in CIA mice after Rg1 therapy. Alcian blue staining results illustrated that mice treated with Rg1 had significantly reduced destruction in the articular cartilage. TRAP and cathepsin K staining results demonstrated a significant reduction of numbers of OCs in the articular cartilage in proximal interphalangeal joints and ankle joints in Rg1-treated mice. In summary, this study revealed that Rg1 reduced the inflammatory destruction of periarticular bone by inhibiting differentiation and maturation of osteoclasts in CIA mice.

## 1. Introduction 

Rheumatoid arthritis (RA) is a chronic autoimmune disease characterised by persistent and symmetric inflammatory polyarthritis of the wrist, finger, knee, ankle, and foot joints. Without effective control of inflammation, RA may lead to irreversible joint destruction. RA has become one of the major diseases leading to disability and can seriously threaten human health [1, 2].

The pathology of RA often results in synovitis and damage to the cartilage and subchondral bone. Core pathological changes occurring in the synovial membrane and histocytology associated with RA include synovial intimal hyperplasia (fibroblast-like and macrophage-like cells), infiltration of inflammatory cells in synovial intima (macrophages cells, lymphocytes, etc.), and angiogenesis in inflamed synovia.

Synovial cells and numerous inflammatory cells in inflammatory joints secrete receptor activator for RANKL, which promotes osteoclast (OC) differentiation and maturation by binding to the RANK receptor; thus, RANKL is a necessary regulator of OC development [3, 4]. RANKL levels are positively correlated with the extent of destruction of the articular cartilage and subchondral bone in RA patients [5]. Therefore, RA-related destruction of bone and cartilage is believed to be primarily mediated by OCs. OCs are oversized multinucleated cells derived from hematopoietic stem cells with a monocyte-macrophage lineage and are the only somatic cells known to present bone-resorbing capacity [6]. Multinucleated cells with the OC phenotype were first discovered by Bromley and Woolley in the synovial tissue of RA patients [7]. In a subsequent research conducted by Suzuki et al., OC-like cells with bone resorption activity were identified by cultivating macrophages, fibroblasts, and lymphocytes from the synovial tissue of RA patients in the absence of external stimuli [8]. Studies on c-Fos mutant mice have also directly proven that OC performs a critical function in bone destruction during inflammatory arthritis. c-Fos mutant mice completely lack functional osteoclasts, whereas other hematopoietic lineages, including T cells, develop normally. Whilst mutant mice [c-Fos(−/−)hTNFtg] develop TNF-dependent arthritis in the absence of osteoclasts, they are fully protected against bone destruction [9]. The aforementioned studies demonstrate that OC precursor cells, OCs, and related factors promoting OC differentiation and maturation are key factors in the induction of inflammatory destruction in the articular cartilage and subchondral bone. The adverse effects of RA are primarily associated with bone destruction, which occurs in early stages of RA. As such, preventing cartilage and subchondral bone destruction is crucial for treating RA [2, 10].

Considering that OCs are important in the process of bone destruction in RA, inhibition of OC differentiation and maturation has become an important therapeutic target. RANKL is a key factor of OC development; denosumab, a therapeutic anti-RANKL monoclonal antibody for RA, has entered phase II clinical trials [11]. Sphingosine-1-phosphate (S1P) regulates the differentiation and maturation of OC precursor cells, and an antagonist of the S1P receptor, FTY720, is able to reduce postmenopausal osteoporosis and bone loss by suppressing the functions of mature OCs. Although FTY720 may potentially be applied in RA treatment [12], research on FTY720 remains at relatively early stages and further clinical studies are required.

Herbal drugs, such as ginsenoside Rg1, present several advantages, including a wide range of pharmacological effects, low toxicity, and multitarget therapeutic effects, although they may had teratogenic effects of rat embryos [13]. These drugs are thus considered breakthroughs in the treatment of RA. Ginsenoside Rg1, a major component of ginseng and* Panax notoginseng*, belongs to the protopanaxatriol family and has various pharmacological effects, including anti-inflammatory analgesic, immunosuppressive and cell proliferation, and apoptosis regulation [14, 15]. Previous studies have confirmed that Rg1 is a plant-derived glucocorticoid receptor agonist and phytoestrogen [16, 17]. Recent reports suggest that extracts from ginseng and* P. ginseng*, such as ginsenoside Rb1, a major ginsenoside metabolite (CK), and an* n*-butanol extract of* P. notoginseng *(BT-201) can inhibit inflammatory responses in animal RA models [18–20]. Rg1 also possesses remarkable anti-inflammatory effects. Recent animal studies have revealed that Rg1 alleviates inflammatory symptoms in mice with collagen-induced arthritis (CIA) and is a potential therapeutic agent for RA [21–24]. However, the mechanism by which Rg1 inhibits inflammatory responses has yet to be defined. The present study explores the therapeutic effects of Rg1 on reducing inflammatory symptoms in mice with CIA by inhibiting OC differentiation and maturation through both* in vitro* and* in vivo* approaches. The results of this work provide a theoretical basis for the treatment of various inflammatory joint diseases, including RA, through the use of herbal drugs.

## 2. Materials and Methods

### 2.1. Chemicals and Reagents

Rg1 was purchased from National Institutes for Food and Drug Control (Beijing, China). The recombinant human receptor activator of nuclear factor kappa-B ligand (RANKL) was purchased from Peprotech (London, UK). *α*-MEM medium, fetal bovine serum, and trypsin were purchased from Gibco (Grand Island, NY, USA). Trizol was purchased from Invitrogen (Grand Island, NY, USA). The TRAP staining kit was purchased from Sigma-Aldrich (St. Louis, MO, USA). The reverse transcription kit was purchased from Takara (Shiga, Japan), and the fluorescence quantitative PCR kit was purchased from Applied Biosystems Inc. (USA). The primers were synthesized by Invitrogen (Grand Island, NY, USA). Osteologic discs were purchased from BD Biosciences (San Diego, CA, USA). The CCK-8 kit was purchased from Dojindo Laboratories (Kumamoto, Japan). The PVDF membrane (microporous film of polyvinylidene difluoride) was purchased from Roche (Switzerland). The BCA protein assay kit was purchased from Pierce in Thermo Fisher Inc. (Rockford, IL, USA). MAPK, IKB, NF-*κ*Bp65, c-Fos, c-Jun, NFATc1, phosphorylated MAPK, phosphorylated IKB, and NF-*κ*Bp65 antibody were purchased from Cell Signaling (Beverly, MA, USA).

### 2.2. Cell Culture

The RAW264.7 murine mononuclear macrophage cell line was obtained from a cell library in the Shanghai Institute of Cell Biology, Chinese Academy of Sciences. The MEM medium for RAW264.7 cell culture was supplemented with 10% FBS, penicillin (100 units/mL), and streptomycin (100 ug/mL). The cells were then placed in a 37°C, 5% CO_2_ incubator. RANKL at a concentration of 50 ng/mL was added to the cell culture to induce OC differentiation in RAW264.7 cells.

### 2.3. Detection of Cell Proliferation

The RAW264.7 cells were inoculated into 96-well plates at a density of 1 × 10^5^/mL (approximately 1000 cells per well in 100 *μ*L of medium). The cells were cultured for 6 h in 100 *μ*L/well of a starvation medium without serum. The serum-free medium was replaced by *α*-MEM containing 10% FBS with or without the presence of Rg1 (1, 10, and 100 *μ*g/mL). The medium was removed after 24, 48, 72, and 96 h of incubation and was replaced by 100 *μ*L of serum-free medium with 10% CCK8 dye. The culture was terminated after 1 h, and the absorbance at 450 nm of each well was measured using a microplate reader. The absorbance value indicates the level of proliferation of cells in each well, and cell growth curves were drawn accordingly.

### 2.4. Detection of Apoptosis via Annexin V-FITC/PI Double Staining

The RAW264.7 cells were inoculated in 6 cm diameter dishes at a concentration of 1 × 10^5^/mL (about 30,000 cells per well in 3 mL of medium) and incubated for 24 h in a 37°C, 5% CO_2_ incubator. The medium was removed, and the cells were cultivated in a fresh medium with or without the presence of Rg1 (1, 10, and 100 *μ*g/mL). The culture medium was replaced every 2 d. After 4 d of incubation, the cells were collected after trypsin digestion and washed twice with ice-cold PBS. The cells were resuspended in 100 *μ*L of Annexin V binding buffer, and 5 *μ*L of Annexin V-FITC solution and 10 *μ*L of propidium iodide (PI) staining solution were then added. The mixture was placed at room temperature in the dark for 15 min, and 400 *μ*L of Annexin V binding buffer was added. All data were detected using a flow cytometer (BD Biosciences, USA) and analyzed using the CellQuest Pro software. Normal cells were negative for both Annexin V and PI staining (lower left quadrant of the cytograms,), the cells in the early apoptotic stage were positive for Annexin V staining but negative for PI staining (lower right quadrant), and cells in the late apoptotic stage or necrotic cells were positive for both Annexin V and PI staining (upper right quadrant). Each experiment was repeated three times.

### 2.5. TRAP Staining of Cells

The RAW264.7 cells were inoculated into 96-well plates at a density of 2 × 10^4^/mL (approximately 2000 cells per well in 100 *μ*L of medium) and cultured with RANKL (50 ng/mL) and with or without varying concentrations of Rg1 (1, 10, 100 *μ*g/mL) for 6 d. The culture medium was replaced with fresh medium containing these reagents every 2 d. The cells in all groups were collected after 4 d of incubation, stained using the TRAP staining kit according to the manufacturer's protocol, and observed using a microscope (100x objective). TRAP-positive multinuclear macrophages with more than three nuclei were counted as OCs. The number of OCs in each well in all groups was counted, and the average of each group was calculated.

### 2.6. Bone Resorption Pit Assay

The RAW264.7 cells were inoculated into osteologic discs at a density of 5 × 10^3^/mL (approximately 500 cells per well in 100 *μ*L of medium) and incubated for 24 h in a 37°C, 5% CO_2_ incubator to induce OC differentiation of the RAW264.7 cells. The wells were divided into a normal control group, a RANKL-stimulated group, and low-, medium-, and high-dose Rg1 groups. The used medium was replaced with a fresh one every 2 d. After culturing for 10 d, the osteologic discs were recovered from the culture, treated with ultrasound to remove the remaining cell debris, and photographed using a microscope (five randomly selected areas per slice). Each photo was analyzed using the Image-J software, and the percentages of the resorbed area of each group were determined accordingly.

### 2.7. Real-Time RT-PCR Analysis

The RAW264.7 cells were inoculated into 6-well plates at a density of 2 × 10^4^/mL (approximately 40 000 cells per well in 2 mL of medium) and incubated for 48 or 96 h in a 37°C, 5% CO_2_ incubator to induce OC differentiation of the RAW264.7 cells. The wells were divided into a normal control group, a RANKL-stimulated group, and low-, medium-, and high-dose Rg1 groups. The used medium was replaced with a fresh one every 2 d, and cells were incubated for 4 d. TRIzol at 1 mL/well was added, and the total cellular RNA was extracted and reverse-transcribed into cDNA by using the Transcriptor First Strand cDNA Synthesis Kit. The cDNAs of the corresponding genes were quantified using the SYBR Green PCR Kit according to the manufacturer's instructions. The primer sequences used were as follows: TRAP: forward: 5′-CCAATGCCAAAGAGATCGCC-3′ and reverse: 3′-TCTGTGCAGAGACGTTGCCAAG-5′; cathepsin K: forward: 5′-GACGCAGCGATGCTAACTAA-3′ and reverse 3′-CCAGCACAGAGTCCACAACT-5′; CTR: forward: 5′-ACCGACGAGCAACGCCTACGC-3′ and reverse 3′-GCCTCACAGCCTTCAGGTAC-5′; MMP-9: forward: 5′-CTGGACAGCCAGACACTAAAG-3′ and reverse: 3′-CTCGCGGCAAGTCTTCAGAG-5′; NFATc1: forward: 5′-CCGTTGCTTCCAGAAAATAACA-3′ and reverse: 3′-TGTGGGATGTGAACTCGGAA-5′; c-Fos: forward: 5′-GCAGAAGGGGCAAAGTAGAG-3′ and reverse: 3′-GTGTATCTGTCAGCTCCCTC-5′; c-Jun: forward: 5′-ACTCGGACCTTCTCACGTCG-3′ and reverse: 3′-TAGACCGGAGGCTCACTGTG-5′. The following PCR program was used: initial denaturation at 95°C for 2 min, 40 cycles of denaturation at 95°C for 1 min, annealing at 55°C for 15 s, and extension at 60°C for 1 min.

### 2.8. Western Blot

The RAW264.7 cells were inoculated into 6 cm diameter dishes at a density of 1 × 10^5^/mL (approximately 300 000 cells per well in 3 ml of medium) and incubated for indicated periods with or without RANKL (50 ng/mL) and/or Rg1 (1, 10, or 100 *μ*g/mL) in a 37°C, 5% CO_2_ incubator to induce OC differentiation. The total protein was extracted and subjected to Western blot. The cells were collected and washed three times by using prechilled PBS. The cells were resuspended in 100 *μ*L of lysis buffer and incubated for 15 min at 4°C. The cell lysate was transferred into a 1.5 mL EP tube and centrifuged at 12 000 r/min for 20 min. The supernatant was transferred to a new EP tube and stored at −80°C or examined immediately for cellular protein content via the BCA method. The levels of P38, phosphorylated P38 (pP38), ERK1/2, phosphorylated ERK (pERK1/2), JNK, phosphorylated JNK (pJNK), I*κ*B*α*, phosphorylated I*κ*B*α* (pI*κ*B*α*), NF-*κ*Bp65, phosphorylated NF-*κ*Bp65 (pNF-*κ*Bp65), NFATc1, c-Fos, c-Jun, and GAPDH were determined as described below. Cell lysates containing 20 *μ*g of total proteins were separated using 15% SDS-polyacrylamide gel electrophoresis (SDS-PAGE) gels and electrotransferred to polyvinylidene difluoride membranes. The membranes were blocked in 5% skim milk in TBS buffer (0.1% Tween-20; pH 7.6) at room temperature for 1 h and incubated with the corresponding primary antibodies overnight at 4°C. After washing with TBS, the membranes were incubated with appropriate peroxidase-conjugated secondary antibodies for 1 h at 37°C with shaking. Immunoreactive proteins were detected using the Molecular Imager ChemiDoc XRS+ Imaging System and Chemiluminescence (Bio-Rad Laboratories Inc., Hercules, CA, USA). The gray values of protein bands were measured using Image Lab 2.0 software (Bio-Rad Laboratories Inc., CA, USA).

### 2.9. Animal Study of CIA Mice

Twenty-four healthy male DBA/1J mice at 6–8 weeks of age (20 ± 2 g) were randomly chosen. Male DBA/1J mice were intradermally immunized at the base of the tail with 200 *μ*g of bovine collagen type II (Chondrex, Redmond, WA, USA) dissolved in 100 *μ*L of 0.05 M acetic acid and mixed with an equal volume of CFA (Difco, MI, USA). Three weeks later, the animals were reimmunized with 200 *μ*g of CII emulsified in incomplete Freund's adjuvant (Difco). Mice were observed twice a day and randomly divided into a collagen solvent group and an Rg1 treatment group (12.5 mg/kg/d) after joint swelling developed (about 28 days after the first injection). Mice in each group (*n* = 8) were given daily intraperitoneal injections for 14 days. Control mice (*n* = 8) were intraperitoneally injected with an equal volume of normal saline. After treatment, the mice were scored based on the degree of joint swelling and the number of affected joints (0–4 points). Details were described as follows: 0 point, no redness and swelling; 1 point, mild redness and swelling in wrist or three toe joints; 2 points, moderate redness and swelling in wrist, ankle, or more than 3 toe joints; 3 points, severe inflammatory reaction in wrist or ankle joints; and points, severe inflammatory reaction in ankle, wrist, and all toe joints. The highest possible score for a mouse was 16. Mice were sacrificed on day 43. Their ankle and toe joints were decalcified with 8% nitric acid solution for 16–18 h followed by conventional dehydration, paraffin infiltration, embedding, cutting (3 *μ*m), baking, and dewaxing. After waxing, synovial hyperplasia, leukocyte infiltration, and cartilage destruction were assessed by hematoxylin-eosin (HE) and Alcian blue staining. The arthritis score (0–4 points) in HE staining was determined as follows: 0 point, normal ankles; 1 point, mild focal infiltration; 2 points, moderate infiltration; 3 points, severe inflammatory infiltration without angiogenesis or cartilage injury; and 4 points, angiogenesis and severe invasion in articular cartilage and subchondral bone. A semiquantitative score in Alcian blue staining was based on a three-point scoring system, where 0 means no loss of proteoglycans and 3 represents complete loss of proteoglycans. Bone slices were baked, dewaxed, and subjected to TRAP and cathepsin K staining as described below. Sections were stained with a tartrate resistant acid phosphatase (TRAP) staining kit (Sigma, St. Louis, MO, USA). In a typical procedure, bone slices were digested by 500 ng/mL pepsin for 5–10 min, treated with 3% hydrogen peroxide for 10 min, and washed three times (2 min each) with PBS containing 0.3% Tween (PBST). Bone slices were then incubated with rabbit anti-mouse cathepsin K antibody (1 : 100 dilution) overnight at 4°C, washed three times with PBST, and incubated with 1 : 200 dilution of horseradish peroxidase- (HRP-) labeled goat anti-rabbit secondary antibodies at room temperature for 1 h. Slices were incubated with DAB for 30 s, rinsed with water for 5 min, mounted, and observed under a microscope. TRAP-positive cells and cathepsin K-positive cells were counted in five areas of each ankle.

### 2.10. Statistical Analysis

All values were expressed as the mean ± SD, unless otherwise stated. Statistical analysis was performed using SPSS 13.0 software. Results from different groups were analyzed by one-way ANOVA followed by Dunnett's post hoc test. Differences with probability (*P*) value less than 0.05 were considered statistically significant.

## 3. Results

### 3.1. Effects of Rg1 on Cell Viability

**Figure 1:**
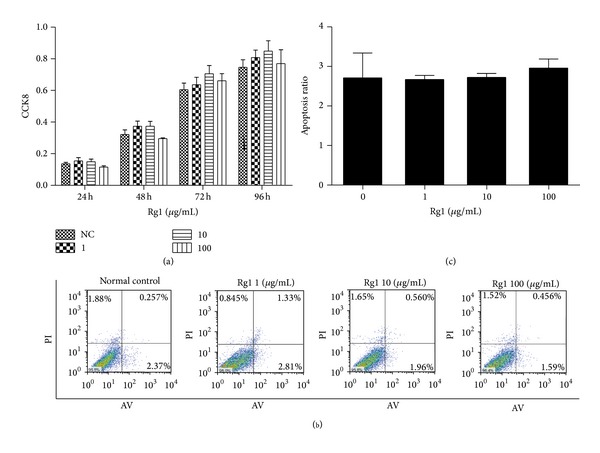
Effect of Rg1 on cell viability and apoptosis. (a) CCK8 assay was performed after incubation of RAW 264.7 cells (2 × 10^4^ cells/mL) with Rg1 (1, 10, and 100 *μ*g/mL) for 24, 48, 72, and 96 h in 96-well plates. (b) RAW 264.7 cells (1.0 × 10^5^ cells/mL) were stimulated with or without the presence of Rg1 (1, 10, and 100 *μ*g/mL) for 96 h in a 6 cm culture plate. Cells were collected and stained with Annexin V and PI and then examined by flow cytometry. (c) FACS histogram of the percentage of early and late apoptosis cells. Values are expressed as the mean ± SD of triplicate experiments. All data are representative of three different experiments.

RAW264.7 cells were cultured with different concentrations of Rg1. Cell proliferation was measured by CCK-8 assay after 24, 48, 72, and 96 h to evaluate the cytotoxicity of Rg1 towards RAW264.7 cells. Cells in the normal control, Rg1 low-dose, medium-dose, and high-dose groups exhibited similar growth curves, and no statistical differences in cellular proliferation were observed amongst these groups. As shown in Figure 1(a), Rg1 at concentrations no greater than 100 *μ*g/mL did not exhibit significant cytotoxicity and did not inhibit cell proliferation. Cells were collected after 96 h of incubation with or without Rg1 (1, 10, and 100 *μ*g/mL) and then stained with Annexin V and propidium iodide (PI) to determine the effect of Rg1 on apoptosis during OC differentiation. The rate of early and late apoptosis in the normal control group was 2.71% ± 0.6285%; by comparison, apoptosis rates in the Rg1 low-dose, medium-dose, and high-dose groups were 2.67% ± 0.106%, 2.723% ± 0.09701%, and 2.957% ± 0.231%, respectively. These results suggest that Rg1 at concentrations no greater than 100 *μ*g/mL has no obvious effect on cell proliferation and apoptosis (Figures 1(b) and 1(c)).

### 3.2. Effect of Rg1 on OC Differentiation in RANKL-Stimulated RAW264.7 Cells

**Figure 2:**
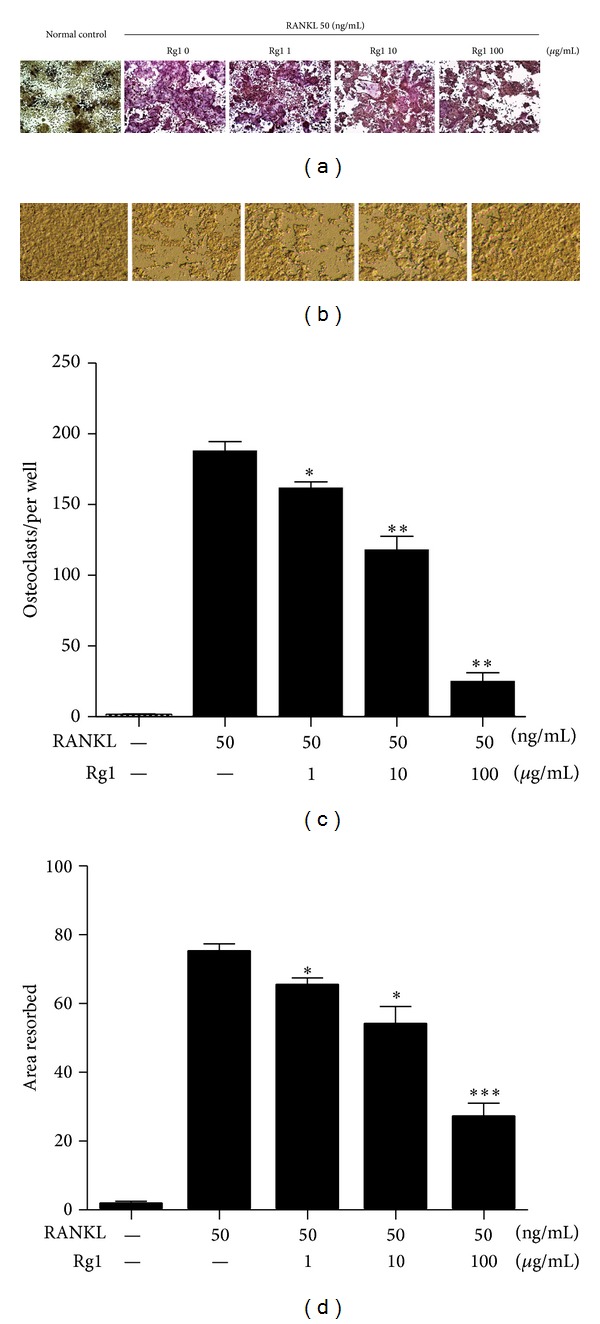
Effects of Rg1 on osteoclast differentiation and resorption pit formation in RANKL-stimulated RAW 264.7 cells. (a) RAW 264.7 cells (2 × 10^4^ cells/mL) were stimulated with RANKL (100 ng/mL) for 96 h with or without the presence of Rg1 (1, 10, and 100 *μ*g/mL). Cells were fixed with 3.7% formalin, permeabilized with 0.1% Triton X-100, and stained with TRAP solution. (b) RAW 264.7 cells (5 × 10^3^ cells/mL) were cultured on bone slices with various concentrations of Rg1 in the presence of RANKL (50 ng/mL). After culturing for 10 days, the dentine slice was recovered from the culture and subjected to visualization of resorption pits. (c) The cells were stained for TRAP, and TRAP-positive multinuclear cells containing more than three nuclei were counted as multinucleated osteoclasts (mOCs). (d) The percentages of resorbed area were determined using Image-J software. Values are expressed as the mean ± SD of triplicate experiments. ∗*P* > 0.05, ∗∗*P* < 0.01, and ∗∗∗*P* < 0.001.

The expression and secretion of TRAP, a marker enzyme for differentiated OCs, are critical to OC differentiation and maturation. TRAP staining is the standard technique for detecting OC formation, and TRAP-positive multinuclear cells containing more than three nuclei are regarded as multinucleated OCs. The effect of Rg1 on OC differentiation and maturation in RAW 264.7 cells was examined through TRAP staining. While no OCs formed in the normal control group, numerous claret-red multinuclear cells were observed in the RANKL-stimulated group. When RANKL-stimulated RAW 264.7 cells were cultured with Rg1 (1, 10, and 100 *μ*g/mL), the number of OCs observed decreased obviously in a dose-dependent manner. Rg1 (100 *μ*g/mL) significantly inhibited OC formation (*P* < 0.01) (Figures 2(a) and 2(c)). These results suggest that Rg1 effectively inhibits OC formation.

OCs are the only known somatic cells with bone-resorbing capacity. The number and area of resorption pits formed on osteologic discs were examined to evaluate the effect of Rg1 on OC differentiation in RAW264.7 cells. As shown in Figures 2(b) and 2(d), osteologic discs in the normal control group are smooth and do not exhibit obvious resorption pits. By contrast, numerous and large resorbed areas were observed in the osteologic discs in the RANKL-stimulated group. Several resorption pits were found in the Rg1 groups (1, 10, and 100 *μ*g/mL), and the number of pits observed decreased in a dose-dependent manner. The areas of the resorption pits were analyzed using Image-J software. The resorbed areas in the Rg1 groups were smaller than those in the RANKL-stimulated group, and Rg1 (100 *μ*g/mL) significantly reduced the area of resorption pits (*P* < 0.01) (Figures 2(b) and 2(d)). In summary, high concentrations of Rg1 dramatically inhibit both the activity and number of OCs and reduce the area of resorption pits.

### 3.3. Effect of Rg1 on the Expression of OC Marker Genes in RANKL Stimulated RAW264.7 Cells

**Figure 3:**
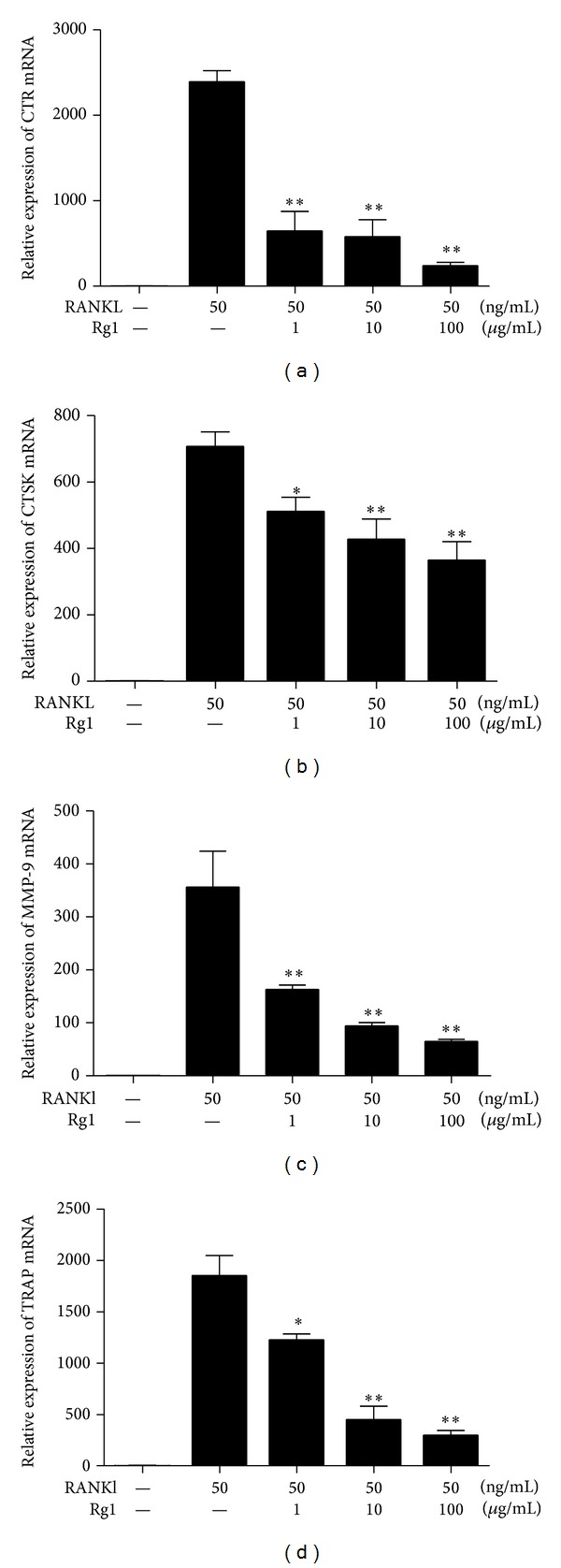
Effect of Rg1 on the mRNA expression of osteoclastic marker genes in RANKL-stimulated RAW 264.7 cells. RAW 264.7 cells (2 × 10^4^ cells/mL) were stimulated with RANKL (50 ng/mL) in the presence of Rg1 (1, 10, and 100 *μ*g/mL) for 96 h in 12-well plates. mRNA expressions of osteoclastogenic marker genes were determined by RT-PCR. Values are expressed as the mean ± SD of triplicate experiments. ∗*P* > 0.05, ∗∗*P* < 0.01, and ∗∗∗*P* < 0.001.

Several proteins associated with OC functions, including Trap, cathepsin K, Ctr, and MMP-9, were expressed during OC differentiation and maturation in RAW264.7 cells. The relative expression levels of Trap, cathepsin K, Ctr, and MMP-9 in the Rg1 groups were quantified through real-time PCR (Figure 3). Results showed that Rg1 significantly inhibits the expression levels of these genes in a concentration-dependent manner.

### 3.4. Effect of Rg1 on the RANKL-Induced Expression of Activation Protein-1 and Nuclear Factor of Activated T Cells in RANKL-Stimulated RAW264.7 Cells

**Figure 4:**
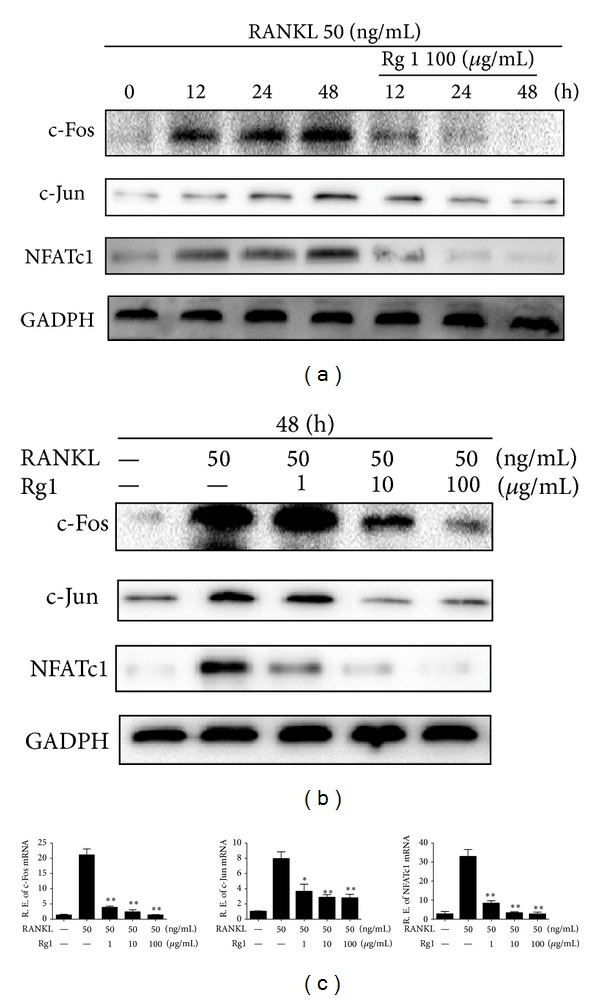
Effect of Rg1 on the RANKL-induced expression of c-Fos, c-Jun, and NFATc1 in RAW 264.7 cells. (a) RAW 264.7 cells (1 × 10^5^ cells/mL) were plated in a 6 cm culture plate and cultured with or without RANKL (50 ng/mL) and/or Rg1 (100 *μ*g/mL) for indicated periods. The cell lysates were subjected to Western blot analysis with an antibody to c-Fos, c-Jun, and NFATc1. (b) RAW 264.7 cells (1 × 10^5^ cells/mL) were plated in a 6 cm culture plate, and RAW264.7 cells were cultured with RANKL (50 ng/mL) and/or increasing concentrations of Rg1 for 48 h. The cells were subjected to Western blot analysis. (c) RAW264.7 cells (2 × 10^4^ cells/mL) were cultured with or without RANKL (50 ng/mL) and/or Rg1 (1, 10, and 100 *μ*g/mL) for 48 h in 12-well plates. The total cellular RNA was extracted and used for RT-PCR analysis to detect c-Fos, c-Jun, and NFATc1 mRNA. All data are representative of three different experiments.

Activation protein-1 (AP-1) and nuclear factor of activated T cells (NFATc1) are essential transcription factors that regulate RANKL-induced OC differentiation and maturation. c-Fos and c-Jun are members of the AP-1 family of transcription factors. Western blot demonstrated that Rg1 inhibits the expressions c-Fos, c-Jun, and NFATc1 associated with OC development (*P* < 0.01) in a dose-dependent manner (Figures 4(a) and 4(b)). The RT-PCR results are consistent with the Western blot findings. As shown in Figure 4(c), Rg1 exerts dose-dependent inhibitory effects on the messenger RNA (mRNA) expressions of the abovementioned genes after 48 h of culture. These results indicate that Rg1 inhibits OC differentiation and maturation by suppressing the expression of transcription factors c-Fos, c-Jun, and NFATc1.

### 3.5. Effect of Rg1 on RANKL-Induced MAPKs and NF-*κ*B Activation in RANKL Stimulated RAW264.7 Cells

**Figure 5:**
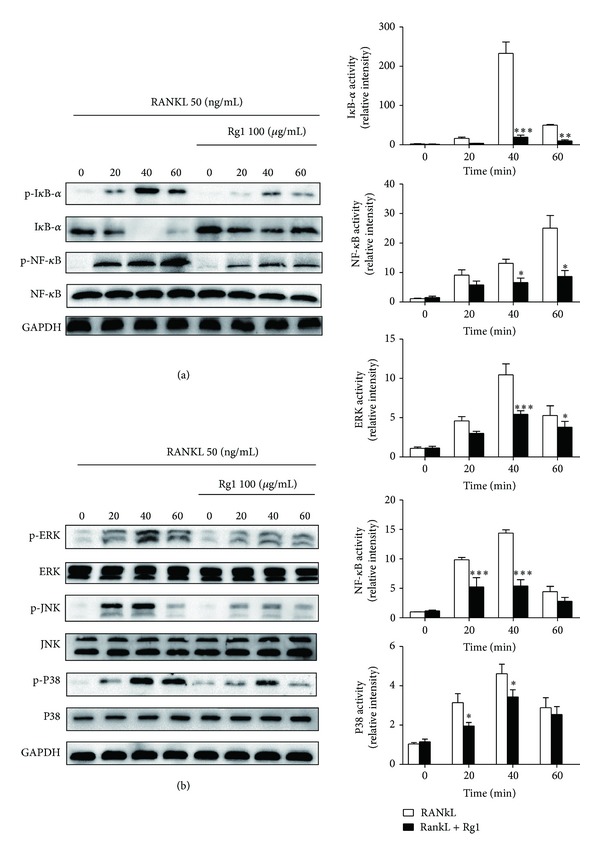
Effects of Rg1 on RANKL-induced I*κ*B, NF-*κ*B, and MAP kinase activation. I*κ*B, NF-*κ*B, and MAP kinase activation was represented by the levels of protein phosphorylation. RAW 264.7 cells (1 × 10^5^ cells/mL) were cultured with or without Rg1 (100 *μ*g/mL) in the presence of RANKL for indicated times. Western blot analysis was performed with whole cell lysates (10 mg). Blots were probed with antibodies specific for I*κ*B, NF-*κ*B, and MAP kinase. The densities of phosphorylated protein (p-) levels (upper panels) were normalized to the density of total protein levels (lower panels). GAPDH was used as the loading control.

Mitogen-activated protein kinase (MAPK) and NF-*κ*B are critical signaling pathways in the process of RANKL-stimulated OC differentiation and maturation. Phosphorylation levels of MAPK, I*κ*B*α*, and NF-*κ*B p65 were measured by Western blot to evaluate the effect of Rg1 on the MAPK and NF-*κ*B signaling pathways. Phosphorylation levels of MAPK and I*κ*B*α* began to increase 20 min after RANKL induction, reaching their peaks at 40 min and gradually decreasing after 60 min. Phosphorylation levels of NF-*κ*B p65 in the RANKL-induced group continued to increase throughout the entire 60 min observation period. However, phosphorylation levels of MAPK (JNK, ERK1/2, and P38), I*κ*B*α*, and NF-*κ*B p65 in Rg1-treated groups were much lower than those in the RANKL-induced group (Figures 5(a) and 5(b)); this finding indicates that Rg1 inhibits the activities of NF-*κ*Bp65, MAPK, JNK, ERK1/2, and P38 during OC differentiation and maturation.

### 3.6. Inhibitory Effect of Rg1 on Arthritis in CIA Mice

**Figure 6:**
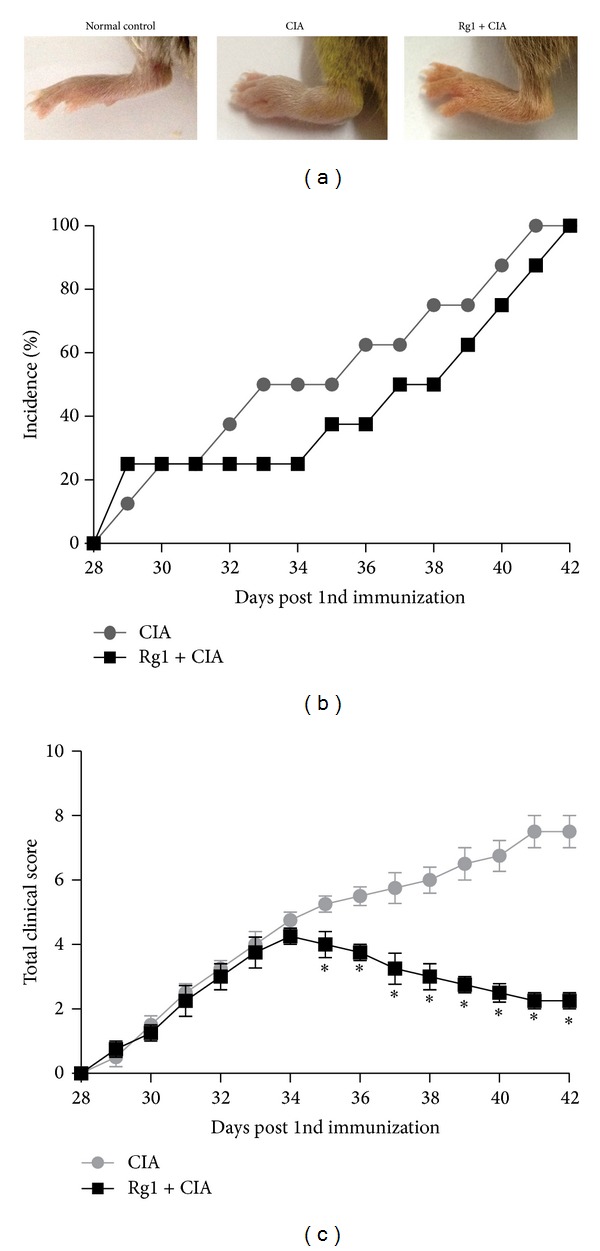
Effects of Rg1 on incidence, arthritis scores, and paw swelling with prolonged time in CIA mice. Mice were intraperitoneally injected with placebo and Rg1 for 14 days after 28 days since the first immunization. Paw thickness (a), incidence (b), and arthritis scores (c) were measured at 1-day intervals. Rg1 markedly reduced the mean arthritis scores and paw swelling. Data are the mean ± SD of eight mice. ∗*P* < 0.05, compared with relevant controls.

Mice were injected intraperitoneally with Rg1 for 14 d on the 28th day after the first immunization. Although the arthritis scores of both CIA and Rg1-treated mice began to increase initially, the scores of Rg1-treated mice were significantly lower than those of CIA mice from days 33 to 42. This result suggests that Rg1 inhibits inflammatory reactions in the joints of CIA mice (*P* < 0.01, Figure 6). Haematoxylin and eosin staining of joint slides indicated that articular capsules in the normal control group feature intact synovial and fibrous membranes. Synovial fluid was completely retained in the joint cavities, and the surfaces of articular cartilages were smooth and without any hyperplasia. By contrast, the synovial membranes of CIA mice were destroyed and infiltration of inflammatory cells was observed in the synovia and synovial tissues. Oedema occurred in tissues adjacent to joints, and joint cavities disappeared.

**Figure 7:**
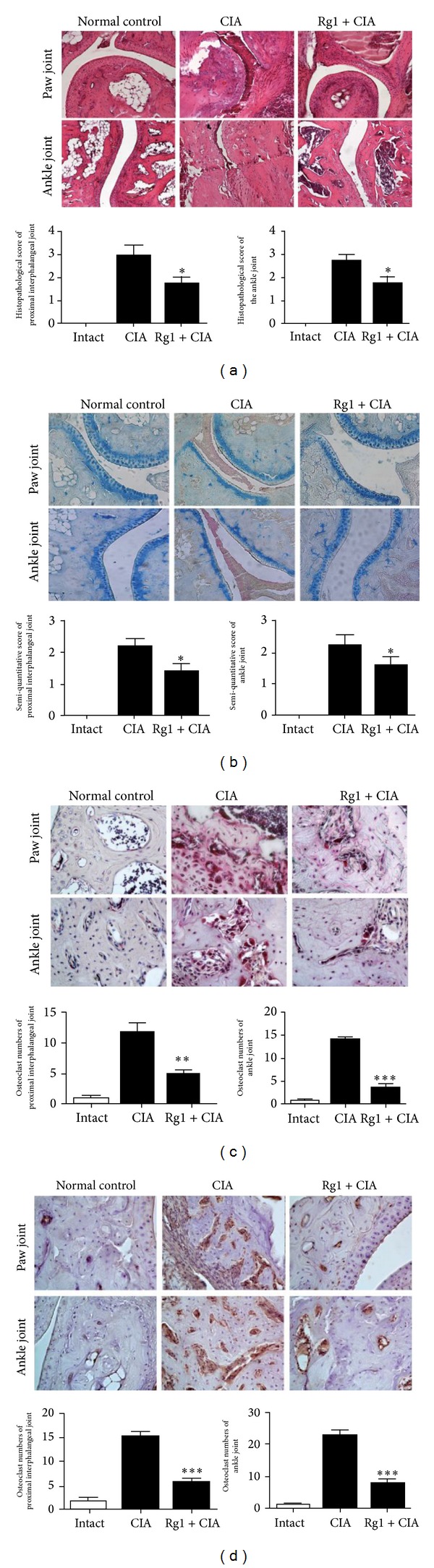
Rg1 resulted in reduced histological inflammation, cartilage loss, and OC formation in CIA mice. (a) Histological evaluations of antiarthritis effects of Rg1 were observed in joint slides stained by HE (original magnification, 200x). Histologic scores in the sections shown in (a). (b) Representative histopathologies of proximal interphalangeal joint, and ankle joint stained with Alcian blue in CIA (original magnification, 200x). Histologic analysis of cartilage in CIA mice after Rg1 therapy. Sections were scored in a blinded manner on a four-point scale as described in Materials and Methods section. A significantly less loss of Alcian blue staining was observed in animals treated with Rg1 than compared to controls, indicating inhibition of cartilage destruction. (c) Trap staining in ankle joints and proximal interphalangeal joint isolated from normal, CIA, and Rg1-treated mice. The expression of Trap significantly decreased in Rg1-treated mice compared with CIA mice. (d) Immunohistochemical staining for cathepsin K in ankle joints and proximal interphalangeal joint isolated from normal, CIA, and Rg1-treated mice. The expression of cathepsin K significantly decreased in Rg1-treated mice compared with CIA mice. Data are the mean ± SD, *n* = 8 mice. ∗*P* < 0.05 versus CIA treated with vehicle.

The extent of destruction in joint capsules in Rg1-treated mice was much less than that in CIA mice. Compared with CIA mice, lower degrees of infiltration of inflammatory cells in the synovia and synovial linings were observed in Rg1-treated mice and the joint cavities of this group increased in size (Figure 7(a)). Histopathological scores of the joints of Rg1-treated mice were significantly lower than those of CIA mice (*P* < 0.01). Histological analysis of joints stained with Alcian blue demonstrated thick cartilage in healthy mice with a smooth surface and without any structural destruction. Compared with health controls, both CIA mice and Rg1-treated mice exhibited different extents of cartilage destruction. Whilst the cartilages of both groups showed rough surfaces, significantly lighter pathological changes were observed in Rg1-treated mice compared with mice in the CIA group; this finding indicates the inhibitory effect of Rg1 on cartilage destruction and inflammatory reactions in joints (Figure 7(b)). To confirm the protective effects of Rg1 against cartilage destruction in CIA mice further, we measured the expression of TRAP and cathepsin K in OCs in murine joints. Whilst TRAP and cathepsin K expressions in both CIA and Rg1-treated mice were higher than those in controls, significantly less TRAP and cathepsin K were expressed in Rg1-treated mice compared with CIA mice (*P* < 0.01, Figures 7(c) and 7(d)), which suggests that Rg1 reduces cartilage destruction by inhibiting OC differentiation and maturation in CIA mice.

## 4. Discussion

The main pathological features of RA include chronic synovial inflammation and cartilage destruction [1, 2, 10]. The goal of antirheumatic treatment is to not only attenuate the clinical symptoms of joint inflammation but also inhibit the progression of joint destruction. Some RA drugs cannot effectively control RA-related bone destruction. For example, prolonged use of methotrexate and dexamethasone leads to further bone destruction by promoting OC differentiation and maturation [25, 26]. An ideal therapeutic drug for RA should efficiently inhibit OC differentiation and maturation and ultimately control bone destruction [11, 12, 27, 28]. Natural compounds are excellent materials for exploring new drug candidates. Ginsenoside Rg1, a major active component of ginseng and* P. notoginseng*, features structural features similar to those of steroid hormones and exhibits various pharmacological effects, including anti-inflammation, antistress activity, and immunoregulation. Amongst these effects, anti-inflammation is the most prominent.* In vitro* experiments demonstrate that Rg1 not only reduces TNF-*α* and IL-6 expression [20] but also inhibits LPS-induced H_2_O_2_ release and decreases CD11b/CD18 expression levels [22].* In vivo* studies show that Rg1 effectively reduces both acute and chronic inflammatory reactions in CIA mice without causing glucocorticoid-induced adverse reactions [24]. Studies show that Rg1 has anti-inflammatory effects* in vivo* and* in vitro*, likely because Rg1 has a rigid steroidal skeleton with sugar moieties, which is the functional ligand of glucocorticoid receptors [16, 24]. Rg1 also has oestrogen-like activities that can reduce glucocorticoid-induced adverse reactions [17]. These two hormone-like effects are probably the basis of the various drug effects of Rg1 and require further study. Rg1 has also been shown to influence bone formation. For instance, Rg1 is able to promote fracture healing in rats [29].* Salvia miltiorrhiza* (Danshen in Chinese) is used to treat osteoporosis because its enriched component, Rg1, has therapeutic effects, such as bone loss inhibition and increased bone formation [30]. However, the mechanisms of the anti-inflammatory and antiosteoporotic effects of Rg1 have yet to be clarified. The current study shows that Rg1 reduces inflammation-related bone destruction by inhibiting OC differentiation and maturation.

OC differentiation and maturation are multistep processes that include cell proliferation, differentiation, fusion, and activation. In the present study, we found that Rg1 inhibits osteoclast differentiation whilst maintaining cell viability. OCs are derived from hematopoietic cells with a mononuclear macrophage cell lineage [6] and the expression of proteins, such as TRAP, CTR, MMP9, and cathepsin K, marks the maturation of large multinuclear OCs with the ability to resorb bones [31–34]. Rg1 dose dependently decreased RANKL-induced expressions of TRAP, CTR, MMP-9, and TSK and reduced bone destruction by inhibiting TRAP activity as well OC numbers. Thus, Rg1 not only significantly inhibits the differentiation of osteoclast precursors into TRAP-positive mononuclear cells but also prevents its fusion to form multinucleated active osteoclasts.

RANKL is an essential factor necessary for osteoclast differentiation and activation. RANKL-deficient mice show a complete absence of osteoclasts and exhibit osteopetrosis [3, 4]. The signaling mechanism of RANKL has been studied extensively. Binding of RANKL to its receptor RANK activates the TRAF protein family, which further induces several signaling pathways, such as those of NF-*κ*B, MAPK, AP1, and NFATcl.

NF-*κ*B transcription factor is essential for RANKL-induced OC development. The classical NF-*κ*B signaling pathway involves activation of the I*κ*B kinase (IKK) complex, which phosphorylates I*κ*B*α* and targets it for ubiquitin-dependent degradation [35]. Previous research demonstrates that p65-deficient mice exhibit high rates of embryonic death whereas mice expressing chimeric p65 manage to survive; these mice have fewer OCs and exhibit remarkably decreased bone resorption after injection of RANKL compared with control mice. PDTC, a specific inhibitor of NF-*κ*B, significantly reduces RANKL-induced TRAP activity in RAW264.7 cells [36]. The available research demonstrates that the NF-*κ*B signaling pathway is crucial for OC differentiation and maturation. Du et al. reported that Rg1 inhibits LPS-induced inflammatory events in RAW264.7 cells by negatively regulating inflammatory mediators and NF-*κ*B activation [24]. Our data showed that Rg1 suppresses activation and nuclear translocation of NF-*κ*B by inhibiting I*κ*B degradation. These results indicate that inhibition of the NF-*κ*B-dependent pathway is one of the mechanisms involved in the antiosteoclastogenic effect of Rg1.

Besides NF-*κ*B, RANKL also activates a series of major intracellular signaling transduction pathways, including those of JNK, ERK, p38, and transcriptional factors, such as AP-1 and NFATc1. RANKL regulates OC differentiation by promoting the activities of three proteins of MAP kinase family, including extracellular signal-regulated kinase (ERK), c-Jun N-terminal kinase (JNK), and p38-MAPK [37, 38]. These kinases also play pivotal roles in the development of osteoclasts and have been considered as key molecular targets for therapeutic application in inflammatory bone destruction [39–43]. In the present study, we evaluated the effects of Rg1 on the activation of these MAPKs and found that Rg1 inhibits their phosphorylation. These results demonstrate that phosphorylation of MAPKs contributes to the antiosteoclastogenic effect of Rg1 in RANKL-stimulated RAW264.7 cells.

NFATc1, a critical factor influencing OC differentiation, regulates the expression of OC marker genes, such as cathepsin k, TRAP, MMP9, and calcitonin receptor [44–46]. AP-1 is composed of Fga (c-Fos, FosB, Fra-I, and Gra-2) and Jun (c-Jun, JunB, and JunD). c-Fos and c-Jun were reported to be critical for transcriptional activation of NFATc1 in RANKL-induced osteoclastogenesis [47–50]. Thus, we propose that inhibition of RANKL-induced c-Fos and c-Jun expression by Rg1 is a relevant factor influencing the suppression of downstream NFATc1 signaling pathways. As a result of downregulation of NFATc1 expression, NFATc1-mediated osteoclastogenic genes, such as TRAP, MMP9, CTR, and cathepsin K [46, 47, 51], which are also concomitantly inhibited by Rg1. Taking these data together, we postulate that Rg1 confers inhibitory activity by inhibiting osteoclastogenesis through downregulation of RANKL-induced NFATc1 expression.

In the current animal experiment, arthritis scores in CIA mice began to obviously decrease after intraperitoneal injection of Rg1 for 6 d. Histopathologies of proximal interphalangeal joints and ankle joints stained with Alcian blue illustrated decreased destruction in the cartilage of CIA mice after Rg1 therapy. TRAP and cathepsin K staining of synovial tissues in CIA mice demonstrated dramatically reduced numbers of OCs after Rg1 treatment. Results from both* in vitro* and* in vivo* experiments reveal that Rg1 adversely affects OC development by suppressing the NF-*κ*B signaling pathway, NFATc1 expression, and MAPK activity, ultimately inhibiting inflammatory responses and cartilage destruction in CIA mice.

Our data suggested that Rg1 markedly inhibited RANKL-induced osteoclast formation in RAW264.7 cells* in vitro*. However, we just examined the direct effect on osteoclast progenitors using RAW264.7 cells that do not require supporting cells. Thus, osteoclast formation in a coculture system could be influenced either by direct effects on osteoclast progenitors or by indirect effects on osteoblasts that support the differentiation of osteoclast progenitors by expressing and secreting RANKL and M-CSF [48]. Thus, we will validate these indirect effects in our further studies by using cocultured primary bone marrow macrophages and osteoblasts. Dexamethasone increases osteoclast formation and lacunar resorption in the presence of M-CSF and RANKL* in vitro* [49]; on the contrary, Rg1, which is the functional ligand of glucocorticoid receptors [16, 24], inhibited osteoclastogenesis in RANKL-induced RAW264.7 cells. This is probably because Rg1 possesses oestrogen-like effects which causes the decrease in osteoclastogenesis [17, 50]. A further study is certainly required.

Simultaneous control of inflammation and cartilage destruction is required for aggressive treatment of RA [1]. Previous research shows that Rg1 is able to suppress inflammation responses in the joints of CIA mice. In the current study, Rg1 not only reduced RA-related inflammation reactions but also inhibited resorption-induced cartilage destruction by inhibiting OC differentiation. Together, our data might decipher the possible molecular mechanisms, by which Rg1 mitigates CIA-evoked bone destruction, and might make Rg1 a potential novel therapy for bone disorders such as RA and osteoporosis by fine-tuning RANKL-induced osteoclast differentiation and functions.

## References

[1] Ngian GS (2010). Rheumatoid arthritis. *Australian Family Physician*.

[2] Silman AJ, Pearson JE (2002). Epidemiology and genetics of rheumatoid arthritis. *Arthritis Research & Therapy*.

[3] Pettit AR, Ji H, Von Stechow D (2001). TRANCE/RANKL knockout mice are protected from bone erosion in a serum transfer model of arthritis. *The American Journal of Pathology*.

[4] Redlich K, Hayer S, Ricci R (2002). Osteoclasts are essential for TNF-α-mediated joint destruction. *Journal of Clinical Investigation*.

[5] Suda T, Takahashi N, Udagawa N, Jimi E, Gillespie MT, Martin TJ (1999). Modulation of osteoclast differentiation and function by the new members of the tumor necrosis factor receptor and ligand families. *Endocrine Reviews*.

[6] Schett G (2007). Cells of the synovium in rheumatoid arthritis: osteoclasts. *Arthritis Research and Therapy*.

[7] Bromley M, Woolley DE (1984). Histopathology of the rheumatoid lesion. Identification of cell types at sites of cartilage erosion. *Arthritis and Rheumatism*.

[8] Suzuki Y, Tsutsumi Y, Nakagawa M (2001). Osteoclast-like cells in an in vitro model of bone destruction by rheumatoid synovium. *Rheumatology*.

[9] Redlich K, Hayer S, Ricci R (2002). Osteoclasts are essential for TNF-α-mediated joint destruction. *The Journal of Clinical Investigation*.

[10] Karmakar S, Kay J, Gravallese EM (2010). Bone Damage in rheumatoid arthritis: mechanistic insights and approaches to prevention. *Rheumatic Disease Clinics of North America*.

[11] Cohen SB, Valen P, Ritchlin C (2006). RANKL inhibition with denosumab reduces progression of bone erosions in patients with rheumatoid arthritis: month 6 MRI results. *Arthritis & Rheumatology*.

[12] Ishii M, Egen JG, Klauschen F (2009). Sphingosine-1-phosphate mobilizes osteoclast precursors and regulates bone homeostasis. *Nature*.

[13] Liu P, Yin H, Xu Y, Zhang Z, Chen K, Li Y (2006). Effects of ginsenoside Rg1 on postimplantation rat and mouse embryos cultured in vitro. *Toxicology in Vitro*.

[14] Jia L, Wang W, Zhou L, Mo F, Li M (2010). Antimotion sickness effects of ginsenosides combined with dexamethasone in rats. *Journal of Chinese Integrative Medicine*.

[15] Radad K, Gille G, Moldzio R, Saito H, Rausch W (2004). Ginsenosides Rb_1_ and Rg_1_ effects on mesencephalic dopaminergic cells stressed with glutamate. *Brain Research*.

[16] Lee YJ, Chung E, Youl Lee K, Hee Lee Y, Huh B, Lee SK (1997). Ginsenoside-Rg1, one of the major active molecules from *Panax ginseng*, is a functional ligand of glucocorticoid receptor. *Molecular and Cellular Endocrinology*.

[17] Chan RYK, Chen W-F, Dong A, Guo D, Wong M-S (2002). Estrogen-like activity of ginsenoside Rg1 derived from *Panax notoginseng*. *The Journal of Clinical Endocrinology & Metabolism*.

[18] Kim HA, Kim S, Chang SH, Hwang HJ, Choi Y (2007). Anti-arthritic effect of ginsenoside Rb1 on collagen induced arthritis in mice. *International Immunopharmacology*.

[19] Chang S, Choi Y, Park J (2007). Anti-inflammatory effects of BT-201, an *n*-butanol extract of *Panax notoginseng*, observed *in vitro* and in a collagen-induced arthritis model. *Clinical Nutrition*.

[20] Choi YS, Kang EH, Lee EY (2013). Joint-protective effects of compound K, a major ginsenoside metabolite, in rheumatoid arthritis: in vitro evidence. *Rheumatology International*.

[21] Rhule A, Rase B, Smith JR, Shepherd DM (2008). Toll-like receptor ligand-induced activation of murine DC2.4 cells is attenuated by *Panax notoginseng*. *Journal of Ethnopharmacology*.

[22] Sun K, Wang CS, Guo J (2007). Protective effects of ginsenoside Rb1, ginsenoside Rg1, and notoginsenoside R1 on lipopolysaccharide-induced microcirculatory disturbance in rat mesentery. *Life Sciences*.

[23] Wu CF, Bi XL, Yang JY (2007). Differential effects of ginsenosides on NO and TNF-α production by LPS-activated N9 microglia. *International Immunopharmacology*.

[24] Du J, Cheng B, Zhu X, Ling C (2011). Ginsenoside Rg1, a novel glucocorticoid receptor agonist of plant origin, maintains glucocorticoid efficacy with reduced side effects. *The Journal of Immunology*.

[25] King TJ, Georgiou KR, Cool JC (2012). Methotrexate chemotherapy promotes osteoclast formation in the long bone of rats via increased pro-inflammatory cytokines and enhanced NF-*κ*B activation. *The American Journal of Pathology*.

[26] Kaji H, Sugimoto T, Kanatani M, Nishiyama K, Chihara K (1997). Dexamethasone stimulates osteoclast-like cell formation by directly acting on hemopoietic blast cells and enhances osteoclast-like cell formation stimulated by parathyroid hormone and prostaglandin E2. *Journal of Bone and Mineral Research*.

[27] Okamoto K, Takayanagi H (2011). Osteoclasts in arthritis and Th17 cell development. *International Immunopharmacology*.

[28] Broadhead ML, Clark JC, Dass CR, Choong PF, Myers DE (2011). Therapeutic targeting of osteoclast function and pathways. *Expert Opinion on Therapeutic Targets*.

[29] Shen Y, Li Y, Li S, Ma L, Ding L, Ji H (2010). Alleviation of ovariectomy-induced osteoporosis in rats by Panax notoginseng saponins. *Journal of Natural Medicines*.

[30] Peng LH, Ko CH, Siu SW (2010). *In vitro* & *in vivo* assessment of a herbal formula used topically for bone fracture treatment. *Journal of Ethnopharmacology*.

[31] Anusaksathien O, Laplace C, Li X (2001). Tissue-specific and ubiquitous promoters direct the expression of alternatively spliced transcripts from the calcitonin receptor gene. *The Journal of Biological Chemistry*.

[32] Motyckova G, Weilbaecher KN, Horstmann M, Rieman DJ, Fisher DZ, Fisher DE (2001). Linking osteopetrosis and pycnodysostosis: regulation of cathepsin K expression by the microphthalmia transcription factor family. *Proceedings of the National Academy of Sciences of the United States of America*.

[33] Reddy SV, Hundley JE, Windle JJ (1995). Characterization of the mouse tartrate-resistant acid phosphatase (TRAP) gene promoter. *Journal of Bone and Mineral Research*.

[34] Choi HJ, Park YR, Nepal M (2010). Inhibition of osteoclastogenic differentiation by Ikarisoside A in RAW 264.7 cells via JNK and NF-*Κ*B signaling pathways. *European Journal of Pharmacology*.

[35] Karin M, Yamamoto Y, Wang QM (2004). The IKK NF-*κ*B system: a treasure trove for drug development. *Nature Reviews Drug Discovery*.

[36] Novack DV (2011). Role of NF-*κ*B in the skeleton. *Cell Research*.

[37] Boyle WJ, Simonet WS, Lacey DL (2003). Osteoclast differentiation and activation. *Nature*.

[38] Lee ZH, Kim H-H (2003). Signal transduction by receptor activator of nuclear factor kappa B in osteoclasts. *Biochemical and Biophysical Research Communications*.

[39] Monje P, Hernández-Losa J, Lyons RJ, Castellone MD, Gutkind JS (2005). Regulation of the transcriptional activity of c-Fos by ERK: a novel role for the prolyl isomerase Pin1. *The Journal of Biological Chemistry*.

[40] Ang E, Liu Q, Qi M (2011). Mangiferin attenuates osteoclastogenesis, bone resorption, and RANKL-induced activation of NF-*κ*B and ERK. *Journal of Cellular Biochemistry*.

[41] Kim HJ, Lee Y, Chang EJ (2007). Suppression of osteoclastogenesis by N,N-dimethyl-D-erythros-phingosine: a sphingosine kinase inhibition-independent action. *Molecular Pharmacology*.

[42] Ikeda F, Nishimura R, Matsubara T (2004). Critical roles of c-Jun signaling in regulation of NFAT family and RANKL-requlated osteoclast differentiation. *The Journal of Clinical Investigation*.

[43] Matsumoto M, Sudo T, Saito T, Osada H, Tsujimoto M (2000). Involvement of p38 mitogen-activated protein kinase signaling pathway in osteoclastogenesis mediated by receptor activator of NF-*κ*B ligand (RANKL). *The Journal of Biological Chemistry*.

[44] Takayanagi H, Kim S, Koga T (2002). Induction and activation of the transcription factor NFATc1 (NFAT2) integrate RANKL signaling in terminal differentiation of osteoclasts. *Developmental Cell*.

[45] Kim Y, Sato K, Asagiri M, Morita I, Soma K, Takayanagi H (2005). Contribution of nuclear factor of activated T cells c1 to the transcriptional control of immunoreceptor osteoclast-associated receptor but not triggering receptor expressed by myeloid cells-2 during osteoclastogenesis. *The Journal of Biological Chemistry*.

[46] Matsumoto M, Kogawa M, Wada S (2004). Essential role of p38 mitogen-activated protein kinase in cathepsin K gene expression during osteoclastogenesis through association of NFATc1 and PU.1. *The Journal of Biological Chemistry*.

[47] Matsuo K, Galson DL, Zhao C (2004). Nuclear factor of activated T-cells (NFAT) rescues osteoclastogenesis in precursors lacking c-Fos. *The Journal of Biological Chemistry*.

[48] Jimi E, Nakamura I, Amano H (1996). Osteoclast function is activated by osteoblastic cells through a mechanism involving cell-to-cell contact.. *Endocrinology*.

[49] Hirayama T, Danks L, Sabokbar A, Athanasou NA (2002). Osteoclast formation and activity in the pathogenesis of osteoporosis in rheumatoid arthritis. *Rheumatology*.

[50] Chen F, Ouyang Y, Ye T (2014). Estrogen inhibits RANKL-induced osteoclastic differentiation by increasing the expression of TRPV5 channel. *Journal of Cellular Biochemistry*.

[51] Asagiri M, Sato K, Usami T (2005). Autoamplification of NFATc1 expression determines its essential role in bone homeostasis. *Journal of Experimental Medicine*.

